# Chronic treatment with fluoxetine regulates mitochondrial features and plasticity-associated transcriptomic pathways in parvalbumin-positive interneurons of prefrontal cortex

**DOI:** 10.1038/s41386-025-02219-8

**Published:** 2025-09-08

**Authors:** Elias Jetsonen, Giuliano Didio, Ilida Suleymanova, Indrek Teino, Eero Castrén, Juzoh Umemori

**Affiliations:** 1 Neuroscience Center, HiLIFE, University of Helsinki, Helsinki, Finland; 2 Faculty of Biological and Environmental Sciences, University of Helsinki, Helsinki, Finland; 3 Institute of Biotechnology, HiLIFE, University of Helsinki, Helsinki, Finland; 4 Gene and Cell Technology, A.I. Virtanen Institute, University of Eastern Finland, Joensuu, Finland

**Keywords:** Inhibition-excitation balance, Cellular neuroscience, Energy metabolism

## Abstract

Chronic treatment with fluoxetine, a widely prescribed selective serotonin reuptake inhibitor (SSRI), is known to promote neural plasticity. The role of fluoxetine in plasticity has been particularly tied to parvalbumin-positive interneurons, a key population of GABAergic neurons that regulate inhibitory tone and network stability. While our previous studies have highlighted fluoxetine-induced plasticity in the visual cortex and hippocampus, its cell-type-specific effects in the prefrontal cortex (PFC) remain unclear. This study aims to investigate the effects of chronic fluoxetine treatment on PV-positive (PV^+^) cells, identified using PV-IRES-Cre-driven reporter expression in the PFC. Using Translating Ribosome Affinity Purification (TRAP), we found that fluoxetine treatment altered the expression of 50 distinct biological pathways. Downregulated pathways are involved in mitochondrial ATP production, including components of the electron transport chain, and ribosomes. Upregulated pathways were associated with phosphatase activity, ion channel function, and cytoskeletal remodeling —molecules broadly implicated in synaptic signaling and plasticity-related processes. In FACS-sorted cells, mitochondrial DNA (mtDNA) expression was significantly increased in PV^+^ cells of the PFC, while intracellular ATP levels remained unchanged. Immunohistochemical analyses demonstrated reduced PV expression and weakened perineuronal nets in specific PFC subregions, suggesting a plasticity-permissive state in PV^+^ cells. TOMM22 signal intensity in PV^+^ cells showed a slight but significant increase in the prelimbic region, suggesting potential compensatory mitochondrial biogenesis despite transcriptomic downregulation of mitochondrial genes. Our findings reveal that chronic fluoxetine induces coordinated transcriptional, structural alterations in PV^+^ cells of the PFC, including shifts in mitochondrial-related gene expression and plasticity-associated pathways. These changes may contribute to region-specific shifts in cortical inhibition and plasticity, complementing previous reports of fluoxetine-mediated behavioral modulation.

## Introduction

Fluoxetine is a widely used antidepressant belonging to the pharmacological group of selective serotonin reuptake inhibitors (SSRI). Preclinical studies have shown that fluoxetine induces juvenile-like plasticity in the adult rodent brain and promotes synaptic remodeling and circuit-level adaption [1–3]. These neuroplastic effects have been observed in diverse neural systems including the visual cortex [4], hippocampus [5] fear-related circuit [6, 7], highlighting its role in modulating brain-wide neuroplasticity. Mechanistically, antidepressants have been shown to directly bind to neurotrophic receptor tyrosine kinase (NTRK2, or TrkB) stabilizing its dimer and enhance BDNF signaling [8].

Parvalbumin positive interneurons (PV-INs), a major class of fast-spiking GABAergic cells, are essential regulators of cortical inhibition and plasticity. They coordinate gamma oscillations [9], synchronize local circuitry [10] and regulate neural plasticity [11]. Our previous studies have shown that PV-INs are central targets of fluoxetine-induced plasticity. Conditional deletion of TrkB in PV-cells reveals that TrkB signaling in these cells is necessary for the behavioral and structural effects of fluoxetine [7, 12].

A key structural feature of mature PV-INs is their envelopment by perineuronal net (PNN), extracellular matrix assemblies that constrain synaptic remodeling and stabilize connectivity, particularly following critical periods of development [13]. Composed of hyaluronan, chondroitin sulfate proteoglycans, and linking proteins [14], PNNs restrict plasticity by limiting AMPA receptor mobility [15], dampening TrkB activation [16], and modulating action potential frequency [17]. Disruption of PNNs or TrkB activation in PV-INs has been shown to shift the excitation-inhibition (E/I) balance, a phenomenon referred to as disinhibition, which supports cortical remodeling in response to antidepressant treatment [12, 18].

PV-INs are also among the most metabolically active neurons, requiring sustained ATP production to maintain fast-spiking activity and inhibitory control [19]. Synaptic activity demands a lot of energy donated by ATP, which is predominantly produced by mitochondria [20]. The role of mitochondria in regulation of E/I-balance and plasticity has been studied in different settings [21, 22]. Interestingly, the inhibition of Complex II of mitochondrial ATP production has been shown to induce LTP in striatal spiny neurons [23]. However, no studies of mitochondrial function, particularly PV-INs, have been published.

Several studies have examined the impact of fluoxetine on mitochondrial function, with conflicting outcomes. For instance, some reports observed increased expression of mitochondrial proteins following fluoxetine [24], while others reported reduced respiration and ATP production, particularly at higher concentrations or after chronic treatment [25–27] These discrepancies likely stem from differences in dosage, treatment duration, or brain region analyzed. Notably, these prior studies were performed at the whole-tissue level, without resolving effects in specific neuronal populations. Whether fluoxetine alters mitochondrial function in PV-INs has not been previously investigated. Given the critical energetic role of mitochondria in PV-INs, we examined this alongside other transcriptional changes to understand how fluoxetine may affect PV-INs physiology and plasticity.

The prefrontal cortex (PFC) is a key hub for cognitive flexibility, emotional regulation, and behavioral control [28]. It integrates signal from limbic and cortical regions including the hippocampus, amygdala, and midbrain [29, 30]. Functionally distinct subregions of the PFC, such as the prelimbic (PL), infralimbic area (IL), and anterior cingulate/motor areas (ACA/M2)—are involved in fear expression, extinction, and adaptive behavior [31–33]. Antidepressants are known to influence PFC connectivity and activation [34], but their cell-type specific effects across PFC subregions remain unclear.

We previously demonstrated that chronic fluoxetine treatment alters gene expression in hippocampal PV positive (PV^+^ ) cells using a translating ribosome affinity purification (TRAP) combined with PV-IRES-cre driver line and identified changes in plasticity- and matrix-related pathways [7]. In the current study, we extend this approach to the PFC, combining TRAP-based transcriptomics, flow cytometry, and subregion-specific immunohistochemistry to investigate the molecular and functional consequences of chronic treatment in PV^+^ cells. We identified a broad transcriptional reorganization in PV^+^ cells of the PFC, including the downregulation of mitochondrial genes and upregulation of pathways related to ion channel activity, intracellular signaling, and extracellular matrix remodeling. To complete the transcriptomic findings, we further assessed mitochondrial DNA and ATP content in FACS-sorting cells. These results suggest that chronic fluoxetine treatment induces a complex shift in PV^+^ cell physiology — impacting not only mitochondrial function, but also plasticity-related signaling and structural pathways across prefrontal cortical circuits.

## Material and methods

### Mice

For the TRAP, transgenic mice expressing FLEX-L10a conjugating GFP specifically in PV^+^ cells were produced by mating females from a homozygous PV-specific Cre line (PV^cre/cre^; Pvalb-IRES-Cre, JAX: 008069, Jackson laboratory) [35] with males from an homozygous mice expressing GFP-L10a [36] (B6;129S4-Gt(ROSA)26Sor ^tm9(EGFP/Rpl10a)Amc^/J, 024750, Jackson laboratory). For PV^+^ cell specific mitochondria analysis, mice were obtained by crossing male transgenic mice harboring double inverted open reading frame (DIO)-expressing tdTomato (B6.Cg-*Gt(ROSA)26Sor*^*tm14(CAG-tdTomato)Hze*^/J, RRID:IMSR_JAX:007914, Jackson laboratory) [37] with female mice of the homozygous PV specific Cre line (PV^cre/cre^) [35].

All mice were kept in a room at 23 ± 2°C with a 12-hr light/dark cycle (lights on at 6:00 a.m.) with access to food and water ad libitum. Three months old male mice are used for this study. All experiments were carried out in accordance with the European Communities Council Directive 86/6609/EEC and the guidelines of the Society for Neuroscience and were approved by the County Administrative Board of Southern Finland (License number: ESAVI/38503/2019).

### Fluoxetine treatment

Fluoxetine (0.008% w/v) was administered in drinking water for 14 days in all experiments. For both control and treated groups, drinking water included 0.1% saccharine for equalizing taste of water. The same treatment conditions were used for all experiments. Water consumption was measured by weighing the bottles before and after being available to the mice (twice per week), and intake was calculated per mouse. As an example, mice in the TRAP experiment consumed approximately 31.0 mg/kg/day of fluoxetine. Supplementary Fig. 1 provides detailed intake measurements.

### TRAP

TRAP analysis was performed according to previously published protocol [38]. The experimental groups consisted of 4 fluoxetine treated and 4 control mice. Briefly, we isolated prefrontal cortex (PFC), including all major subregions (e.g., PL, IL, ACA, M2), from mice expressing GFP tag in ribosomes of PV^+^ cells, as mentioned above. The isolated PFC were stored in -80 C between isolation and TRAP analysis. After lysating the PFC, the GFP-tagged ribosomes were precipitated using magnetic beads coated with anti-GFP antibodies. Quality of RNA was assessed by Bioanalyzer (Agilent, California, US), and one sample from fluoxetine group was excluded due to low RNA integrity (2.4 while it was between 7.6 and 9.5) (Supplemental Fig. 2). Actively translated mRNA co-precipitated and were sequenced using HiSeq2500 (Illumina, CA, USA) with SMART-Seq v4 Ultra Low Input RNA kit (Takara, Japan) for making cDNA library. Precipitation efficiency was confirmed by comparing parvalbumin mRNA expression of one sample before and after precipitation (Supplemental Fig. 3).

### FACS-based assay of ATP and mitochondrial DNA and in PV^+^ cells

PV-TdT mice were anesthetized using pentobarbital and transcardially perfused with cooled N-methyl-D-glucamine buffer [39] to preserve cells. Brains were isolated, and hippocampus, prefrontal cortex and rest of the cortex were dissected. Each experimental group (control and fluoxetine-treated) consisted of four mice (*n* = 4). Samples were dissociated into single-cell suspensions using Neural Tissue Dissociation Kit (Miltenyi, Germany) according to manufacturer’s protocol. Cell suspensions were filtered through 35 μm strainers, and red fluorescent PV^+^ cells (tdTomato +) and non-PV (tdTomato −) cells were isolated by were then fluorescence-activated cell sorting (FACS). Gating strategy for FACS is presented in Supplemental Fig. 4.

Using the sorted cells, ATP was detected using CellTiter Glo 2.0 kit (Promega, Wisconsin US) following the manufacturer’s protocol. Briefly, sorted cells were collected into 96-well white opaque plates, and an equal volume of CellTiter-Glo reagent was added. Plates were shaken for 2 min to induce cell lysis and incubated at room temperature for 10 min to stabilize the luminescent signal. Luminescence was measured using a Varioskan Flash plate reader (Thermo Fisher, Massachusetts, USA).

For mitochondrial DNA (mtDNA) quantification, sorted cells were pelleted by centrifugation at 30,000 x g for 10 min at 4 °C and stored at -20 C until analysis. DNA was extracted using the NucleoSpin Tissue XS DNA extraction kit (Macherey-Nagel, Germany). Mitochondrial DNA levels were quantified by qPCR using primers targeting mt-Rnr1 and normalized to the nuclear gene Rbm15. PCR reactions were run in triplicates using CFX Opus 96 (Bio-Rad, California, US) and SYBR green 2x mix (Thermo Fischer, Massachusetts, US) according to manufacturer’s protocol. All reactions. Quantification was done using delta-Cq method, and expression of mtDNA gene mt-Rnr1 was compared to genomic gene Rbm15. Primer sequences are listed in Supplemental note.

### Immunohistochemistry for PV, PNN and TOMM22

Mice were deeply anesthetized and perfused with 4% PFA in PBS. The brains were cut into 40 µm sections and stored in +4 °C until further processing. Sections were then washed with PBST and blocked using bovine serum albumin and donkey serum. For PV-IRES-Cre line validation, sections from PV-tdTomato mice were processed for double immunostaining with primary antibodies against PV and SST. For functional analyses, triple immunostaining was performed using primary antibodies against PV, PNN, and TOMM22, incubated overnight at 4 °C. After washing, sections were incubated with fluorescently labeled secondary antibodies for 1 h at room temperature. Details of used primary and secondary antibodies and their dilutions are listed in Supplemental Table 1. Images were obtained using a LEICA THUNDER IMAGER 3D TISSUE slide scanner (Leica Microsystems, Germany) for PV-IRES-Cre line validation and an Andor Dragonfly (Oxford Instruments, United Kingdom) for PV, PNN, and TOMM22 functional analyses.

### Imaging analysis

The entire medial prefrontal cortex (mPFC) was imaged as Z-stacks across multiple tiled frames. Images were stitched in ImageJ, and the optimal focal plane was manually selected for each field. Subregions (PL, IL and ACA/M2) were delineated using a standard mouse brain atlas [40]. PV^+^ cells were automatically identified using custom MATLAB scripts combining morphological feature analysis and intensity thresholding. Identified PV^+^ cells were categorized into low, middle, high, and very high PV expression groups. TOMM22 and PNN intensities were quantified separately within each PV expression subgroup. Only PV-positive cells were included in the TOMM22 and PNN analyses to avoid contributions from non-PV cells. Statistical analysis for immunohistochemistry data was conducted at the individual cell level (i.e., each cell was treated as a data point), and degrees of freedom reflect the total number of analyzed cells. A minimum of *n* = 4 mice per group was used, and summary statistics are reported in the figure legends. For PV-IRES-Cre line validation, tdTomato-positive cells in PV-tdTomato mice sections were analyzed for colocalization with PV and SST immunostaining using the same intensity-based analysis methods described above in the whole PFC region. For this validation experiment, *n* = 3 mice were used and colocalization ratios were calculated by averaging across animals.

### Experimental design and statistical analysis

Statistical analysis for TRAP was conducted using R and package DESeq2 [41]. Multiple test correction was performed using Benjamini-Hochberg procedure, and *q*-value (corrected *P*-value) of 0.1 was considered significant. Detected differentially expressed genes (DEGs) are listed in Supplemental Table 2. Pathway analysis was performed using package clusterProfiler [42]. Resulting pathways were clustered and visualized using the function “emapplot_cluster”. All detected pathways are listed in Supplemental Table 3.

Data from immunohistochemical, mitochondrial membrane potential, mitochondrial mass, and ATP assays were analyzed by two-way ANOVA followed by post hoc test with multiple test correction using Šídák’s method, or Chi-square test when suitable. Statistical analyses were performed using Prism 9 (GraphPad Software, California, US). *P*-value < 0.05 was considered significant. Statistical details of these tests are listed in Supplemental Table 4.

## Results

### Identification of PV^+^ cell specific gene expression changes following chronic fluoxetine treatment

**Fig. 1:**
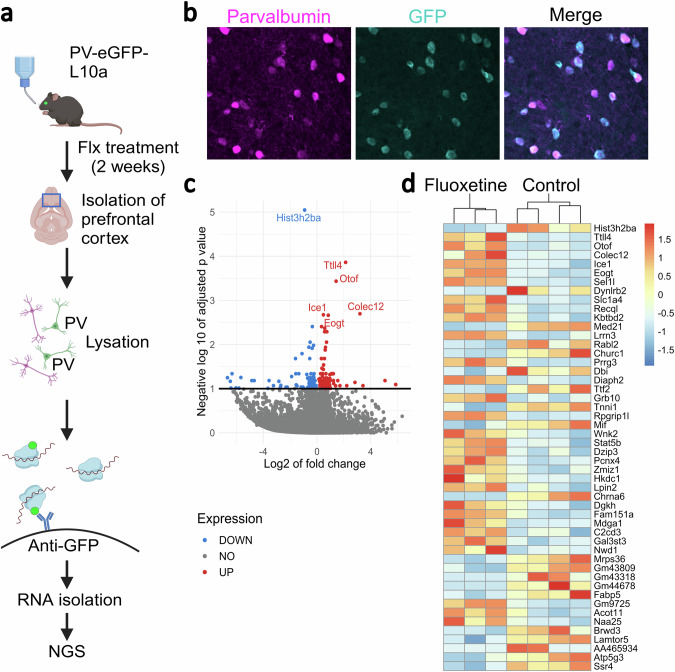
Fluoxetine-induced transcriptional changes in PV^+^ cells from the PFC. **a** PV-eGFP-L10a mice were treated with fluoxetine or regular water for 2 weeks, and their PFC were isolated post-euthanasia. GFP-tagged translating ribosomes binding mRNA were precipitated from cell lysate using magnetic beads coated with anti-GFP antibodies, enabling the isolation of ribosome-bound mRNA. The isolated mRNA was then converted into cDNA and sequenced using next-generation sequencing (NGS). **b** Representative PFC section from PV-eGFP-L10a mouse stained with parvalbumin antibody. GFP co-localizes with parvalbumin, confirming the specificity of the PV^+^ cell labeling **c** Volcano plot showing differentially expressed genes in PV^+^ cells, with the x-axis representing the magnitude of change (log2 fold change) and the y-axis representing statistical significance ( − log10 adjusted *p*-value) on. Upregulated genes are highlighted in red and downregulated genes in blue, providing a visual overview of the transcriptional changes induced by fluoxetine. **d** Heatmap of 50 most differentially expressed genes (DEGs) in PV^+^ cells after chronic fluoxetine treatment, illustrating the extent and pattern of gene expression changes.

After two weeks of fluoxetine treatment, we performed Translating Ribosome Affinity Purification (TRAP) analysis on PV^+^ cells (Fig. 1a, b). Following the application the Benjamini-Hochberg procedure for multiple test correction, we identified 315 DEGs (q < 0.1) (Fig. 1c, Supplemental Table 2). The top 50 DEGs with the lowest *q*-values are shown in Fig. 1d.

**Fig. 2:**
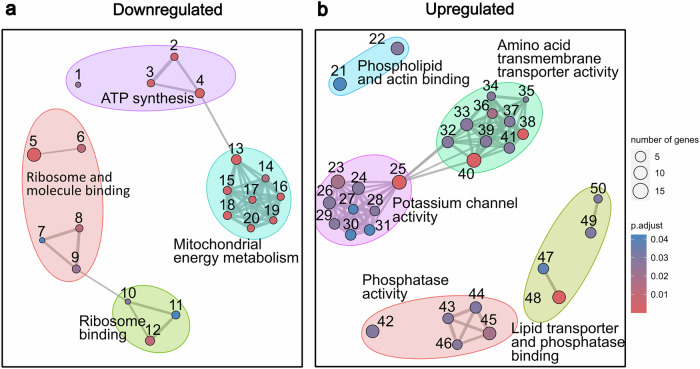
TRAP Analysis in PV^+^ cells after Chronic Fluoxetine Treatment. **a, b** Results of pathway enrichment analysis of GO Molecular Function -pathways in PV^+^ cells clustered into 50 subgroups. Numbers correspond to individual pathways in Supplementary Table 3. **a** Downregulated pathways predominantly involve mitochondrial energy production, including NADH dehydrogenase activity, oxidoreductase activity, cytochrome c function, electron transport, proton transmembrane transport, and ATP synthase. Additionally, downregulation of ribosome-related pathways is observed, suggesting a broad impact on cellular metabolism and protein synthesis. **b** Upregulated pathways are related to phosphatase activity, amino acid transmembrane transport, and voltage-gated potassium channels, highlighting enhanced synaptic and signaling activities [12]. The complete details of the 50 pathways are listed in Supplemental Table 3.

**Fig. 3:**
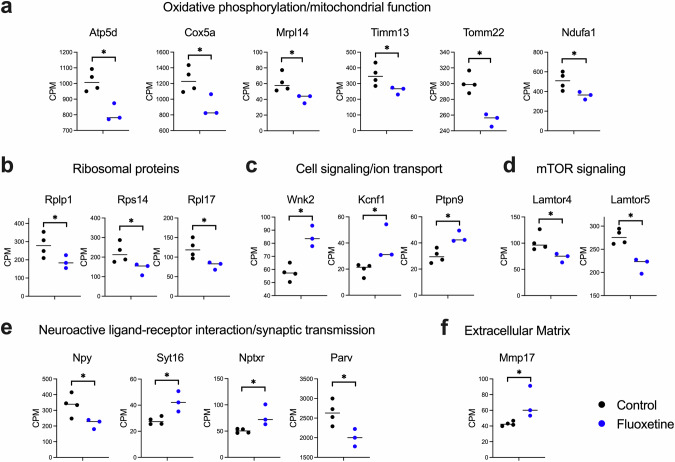
Representative DEGs After Chronic Fluoxetine Treatment. **a** Oxidative Phosphorylation and Mitochondrial Function: Downregulated genes Atp5d, Cox5a, Mrpl14, Timm13, Tomm22, and Ndufa1 are critical components of the mitochondrial electron transport chain, protein synthesis and transmembrane transport. **b** Ribosomal Proteins: Downregulated genes Rplp1, Rps13, and Rpl17 are involved in ribosome biogenesis and function, indicating a potential decrease in protein synthesis capacity. **c** Cell Signaling/Ion Transport: Upregulated genes Wnk2, Kcnf1, and Ptpn9 are associated with ion transport and signaling pathways. **d** mTOR Signaling and Lysosomal Function: Downregulated genes Lamtor4 and Lamtor5 are part of the mTOR signaling pathway and lysosomal function. **e** Neuroactive Ligand-Receptor Interaction/Synaptic Transmission: Downregulated genes Npy and Parv suggest reduced inhibitory signaling and calcium buffering. Upregulated genes Syt16 and Nptxr indicate enhanced synaptic transmission and receptor clustering. **f** PNN interaction: Mmp17, an upregulated gene, plays a key role in matrix remodeling and may lead to the degradation of perineuronal nets (PNNs). Receptor type protein tyrosine phosphatase (Ptprs) was upregulated. It modulates TrkB activity in interaction with PNNs. * indicates statistical significance with *q* < 0.1.

We then conducted a pathway enrichment analysis using Gene Ontology (GO) Molecular Function -pathways for these DEGs and identified upregulation in 30 and downregulation of 20 functional pathways (Fig. 2a, b, Supplemental Table 3). Key representative DEGs in these pathways are shown in Fig. 3. Notably, among the downregulated pathways, several pathways were related to mitochondrial function, including respiratory chain, NADH dehydrogenase (quinone) activity, respiratory chain complex I assembly, mitochondrial transmembrane transport, and mitochondrial ribosome complex (Fig. 2a), where the downregulated genes included Atp5d, Cox5a, Mrpl14, Timm13, Tomm22, and Ndufa1 [43, 44].

We also observed significant downregulation in ribosome-associated pathway, including Rplp1, Rps13, and Rpl17 (Fig. 3b), which are components of the large and small ribosomal subunits [45, 46].

The upregulated pathways were associated with phosphatase activity, amino acid transmembrane transport, voltage gated potassium channel activity, and ion channel activity (Fig. 2b). Representative upregulated genes included Wnk2 and Ptpn9 (Fig. 3c), which are known to be involved in ion homeostasis and cellular signaling [47, 48]. Actin binding related genes were also upregulated, as lipid transporters. We also identified upregulation of the voltage-gated potassium channel gene Kcnf1 (Kv5.1), which encodes a modulatory subunit of potassium channels complex [49].

In addition, genes associated with mTOR signaling and lysosomal function, such as Lamtor4 and Lamtor5 were downregulated (Fig. 3d) and genes encoding neuropeptides and calcium-binding proteins, such as Neuropeptide Y (Npy) and Parvalbumin (Parv) were also downregulated (Fig. 3e). Among the upregulated genes, we identified Synaptotagmin 16 (Syt16) and Neuronal Pentraxin Receptor (Nptxr), which are involved in synaptic vesicle cycling and receptor localization at synapses respectively [50].

Lastly, genes related to extracellular matrix regulation and perineuronal net interaction were also found to be upregulated (Fig. 3f). This includes Matrix metalloproteinase-17 (Mmp17), involved in matrix modeling [51] and Ptprs, a receptor interacting with chondroitin sulfate proteoglycans is also known to be involved in memory retention [16].

Altogether, these transcriptional changes affected diverse molecular functions in PV^+^ cells, spanning mitochondrial metabolism, translational regulation, ion homeostasis, synaptic transmission, and extracellular matrix remodeling.

### Chronic fluoxetine treatment does not alter ATP levels but increases mitochondrial DNA in PV^+^ cells

**Fig. 4:**
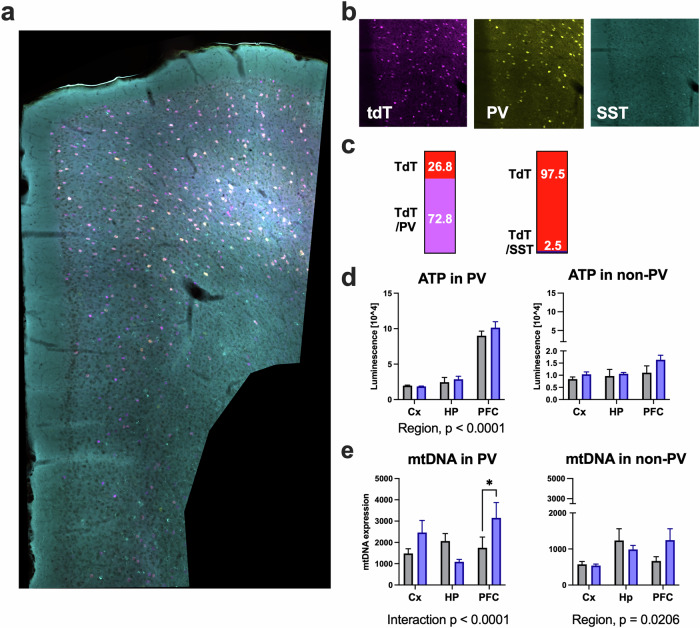
Specificity of PV-tdTomato labeling and mitochondrial assays in FACS-sorted cells from cortex (Cx), hippocampus (HP) and prefrontal cortex (PFC). **a** Stitched confocal image of a coronal PFC section from a PV-IRES-Cre × Ai14 mouse showing tdTomato-positive cells (magenta) with parvalbumin (PV, yellow) and somatostatin (SST, cyan) immunostaining. **b** High-magnification single-channel views demonstrating colocalization patterns (tdTomato, magenta; PV, yellow; SST, cyan, scale bar 100 μm). **c** Quantitative specificity analysis of tdTomato labeling. Left: 72.8% of tdTomato+ cells co-express PV, while 26.8% are PV-negative. Right: Only 2.5% of tdTomato+ cells co-express SST, confirming high specificity for PV lineage (*n* = 3 mice, 730 cells analyzed). **d** Intracellular ATP levels in PV^+^ and non-PV cells across brain regions. PV^+^ cells show significant regional differences (two-way ANOVA, Region F(2,12) = 120.6, *p* < 0.0001) with no fluoxetine treatment effect. Non-PV cells show no significant effects. e) Mitochondrial DNA copy number in sorted cell populations. PV^+^ cells display Treatment × Region interaction (F(2,60) = 4.612, p = 0.0137) with post-hoc increase in PFC after fluoxetine (*p = 0.0271, Fisher’s LSD). Non-PV cells vary by region (F(2,66) = 4.119, *p* = 0.0206) with no treatment effect. Data represents mean ± SEM. Gray bars: vehicle control, blue bars: fluoxetine treatment. * *p* < 0.05 for post-hoc comparisons.

To evaluate the impact of chronic fluoxetine treatment on mitochondrial energy regulation, we assessed intracellular ATP levels and mitochondrial DNA expression in FACS-sorted PV^+^ cells and non-PV cells isolated from PV-tdTomato (PV-tdT) mice (PV-IRES-Cre;Ai9) [35, 37]. First, to validate the specificity of the PV-IRES-Cre line in the prefrontal cortex, we conducted immunohistochemistry using anti-PV and anti-SST antibodies. Our quantitative analysis confirmed that 72.8% of tdTomato-positive cells were PV-immunoreactive, while only 2.5% co-expressed SST protein (Fig. 4a–c). This demonstrates substantial overlap between PV expression and Cre-mediated reporter labeling in the PFC, with minimal off-target labeling in SST-positive interneurons.

No significant changes in ATP levels were detected in any cell type, following fluoxetine treatment (Fig. 4a, c). However, ATP levels were significantly higher in the PFC compared to the hippocampus and other cortical regions, specifically in PV^+^ cells (*p* < 0.0001), regardless of treatment.

Two-way ANOVA for the mitochondrial DNA level was significant for “Interaction” in PV^+^ cells (*p* = 0.0137) and “Region” in non-PV cells (*p* = 0.0206). Post hoc analysis (Fisher’s LSD test) showed that the mitochondrial DNA level was significantly increased in PV^+^ cells (*p* = 0.0271) in the PFC (Fig. 4b, d). No significant effect of fluoxetine was observed in other regions. These results indicate that fluoxetine increases mtDNA expression selectively in the PFC while intracellular ATP level remains unchanged.

### Chronic fluoxetine modulates PV Cell distribution and PNN structure in the PFC

**Fig. 5:**
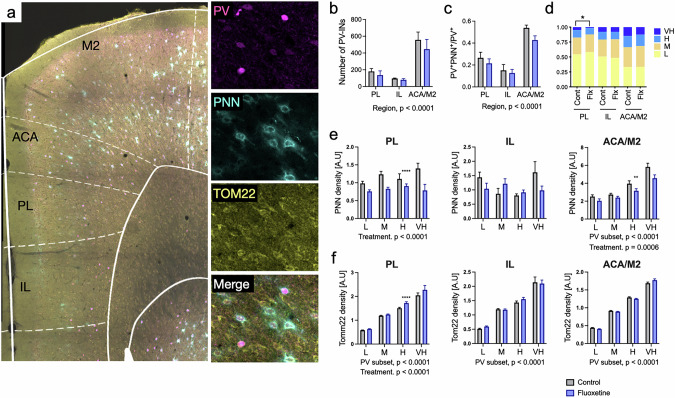
Immunostaining and imaging analysis PV, PNN and TOMM22 in the PFC. **a** Immunostaining and imaging analysis were performed to assess parvalbumin (PV), perineuronal nets (PNN), and TOMM22, a quantitative mitochondrial marker. Sub-regions of the prefrontal cortex, including the infralimbic region (IL), prelimbic region (PL), and anterior cingulate area with secondary motor cortex (ACA/M2), were identified using a reference atlas and selected for subsequent analysis. **b** Number of PV^+^ cells in different subregions. Fluoxetine treatment did not have a significant effect on cell count. However, significant regional differences are observed (F (2, 18) = 22.53, p < 0.0001). **c** The proportion of PV^+^ cells surrounded by PNN. Although no significant treatment effect was observed, the ratio of PNN-positive PV^+^ cells varied significantly among regions (two-way ANOVA, Region, F (2, 18) = 36.35, *p* < 0.0001). **d** PV^+^ cells were categorized into four subgroups based on PV expression levels. The relative number of cells expressing very high levels of PV was significantly lower in the PL region following fluoxetine treatment (Chi-square test, *p* = 0.0175). **e** The intensity of PNNs in PV-cell subsets (low, middle, high, and very high PV expression) was analyzed after chronic fluoxetine treatment in the PL, IL, and ACA/M2 regions. In both the PL and ACA/M2 regions, fluoxetine treatment significantly reduced the overall intensity of PNNs (Two-way ANOVA, Treatment, PL: F (1, 335) = 17.28, *p* < 0.0001; ACA/M2: Treatment, F (1, 1744) = 11.68, *p* = 0.0006). Additionally, there was a significant effect of PV expression subsets (Two-way ANOVA, PV subset, ACA/M2: F (3, 1744) = 40.64, *p* = 0.0006), indicating a positive correlation between PNN intensity and PV expression in the ACA/M2 region. **f** The intensity of TOMM22 in PV-cell subsets. In all regions, a significant effect of PV expression subsets was observed (Two-way ANOVA, PV subset, PL: F (3, 1287) = 791.0 *p* < 0.0001; IL: F (3, 714) = 390.7, *p* < 0.0001; ACA/M2: F (3, 4012) = 2756, *p* < 0.0001), indicating that PV^+^ cells with higher PV expression exhibited higher TOMM22 levels compared to those with lower PV expression. Fluoxetine treatment significantly increased TOMM22 expression only in the PL region (Two-way ANOVA, Treatment, F (1, 1287) = 21.17, *p* < 0.0001). Bars represent standard error of means, with ANOVA results noted below each plot. **, *p* < 0.01; ****, *p* < 0.0001.

To investigate plastic changes and mitochondrial distribution in specific subregions of the prefrontal cortex (PFC), we performed immunohistochemical analysis staining with antibodies of PV and PNN, plastic markers of PV^+^ cells, and TOMM22, a marker of mitochondrial mass and function (Fig. 5a). For TOMM22 analysis, we specifically measured the fluorescence intensity only within PV^+^ cells to evaluate mitochondrial changes specifically in this neuronal population. This analysis excluded TOMM22 signal from surrounding non-PV cells, allowing us to observe cell type-specific effects. The analysis focused on the prelimbic area (PL), infralimbic area (IL), and anterior cingulate area with supplementary motor area (ACA/M2), based on a reference atlas [40]. There was no treatment effect of fluoxetine on number of PV^+^ cells, or the proportion of PV^+^ cells surrounded by PNNs (Fig. 5b, c). However, significant sub-regional differences in both the number of PV^+^ cells and the ratio of PNN-positive PV^+^ cells were observed across the subregions (Fig. 5b, c).

PV-positive cells were categorized into four subgroups based on PV expression levels, as previous studies have shown a negative correlation between PV expression intensity and plasticity in PV^+^ cells [7, 11, 12]. Chronic fluoxetine treatment led to a significant decrease in the number of cells expressing very high levels of PV, particularly in the PL region (*p* = 0.0175) (Fig. 5d), which is consistent with previous findings in the visual cortex [12] and hippocampus [7]. The analysis of PNNs revealed a significant reduction in total PNN intensity in both the PL and ACA/M2 regions following fluoxetine treatment (PL, *p* < 0.0001; ACA/M2, *p* = 0.0006). Additionally, there was a significant effect of PV expression subsets, with a positive correlation between PNN intensity and PV expression levels in the ACA/M2 region (*p* = 0.0006) (Fig. 5e). Two-way ANOVA revealed a slight but statistically significant increase in TOMM22 intensity across PV^+^ cell subsets in the PL region following fluoxetine treatment (*p* < 0.0001) (Fig. 5e, PL panel). In all regions, TOMM22 intensity was positively correlated with PV expression levels, with higher PV-expressing PV^+^ cells exhibiting higher TOMM22 signal (*p* < 0.0001).

These results suggest that chronic fluoxetine treatment induces a plasticity-permissive state in PV^+^ cells and alters PNN structure, particularly in the PL and ACA/M2 regions. The reduction in the number of high PV-expressing PV^+^ cells and corresponding PNN intensity implies increased plasticity in these regions. While fluoxetine did not cause a consistent or large-scale change in TOMM22 expression, the observed correlation with PV levels may reflect underlying differences in metabolic demand between PV^+^ cell subtypes.

## Discussion

### Fluoxetine-induced molecular and structural reorganization in PV^+^ cells of the PFC

The presented findings reveal that chronic fluoxetine treatment induces coordinated transcriptional and structural changes in PV^+^ cells of the prefrontal cortex. Our TRAP analysis identified downregulation of genes associated with mitochondrial function and energy metabolism, and protein translation, alongside upregulation of genes involved in ion channel activity, intracellular signaling, and cytoskeletal remodeling. These shifts suggest a transition toward a state supportive of synaptic reorganization.

Immunohistochemical data further revealed reduced PV expression and perineuronal net (PNN) intensity in specific PFC subregions, including the PL and ACA/M2, which are functionally relevant for emotional and cognitive regulation. While these features have been linked to enhanced plasticity in previous work, in this study, they serve as structural correlates of the transcriptomic reprogramming we observed.

Importantly, mitochondrial DNA levels were increased without corresponding changes in ATP levels, indicating that mitochondrial regulation occurs at the gene expression and DNA content level. Together, these data suggest that chronic fluoxetine promotes a plasticity-permissive state in PV^+^ cells, characterized by molecular downscaling of energy and translational pathways and concurrent upregulation of mechanisms that support synaptic remodeling.

By focusing on cell-type–specific transcriptomic analysis using TRAP methodology in the adult PFC, this study provides new insight into how fluoxetine modulates cortical inhibitory circuits at the molecular and structural levels.

### Implications of transcriptional regulation in PV^+^ cells after chronic fluoxetine

Our TRAP analysis revealed that chronic fluoxetine treatment leads to a broad and complex reorganization of genes expression in PV^+^ cells of the PFC. One of the most striking features was the downregulation of genes associated with mitochondrial function. These included key components of the electron transport chain (Atp5d, Cox5a, Ndufa1), mitochondrial protein import machinery (Tomm22, Timm13), and mitochondrial ribosomal subunits (Mrpl14). Such a coordinated downregulation suggests altered mitochondrial oxidative phosphorylation, which may affect ATP synthesis capacity and availability in PV^+^ cells. Given the high metabolic demands of PV-INs [19], this suppression may contribute to a lowered excitatory state or reduced firing fidelity, as previously demonstrated [12].

In addition to mitochondrial changes, we observed reduced expression of ribosomal protein genes (e.g., Rplp1, Rps13, Rpl17), indicating decreased capacity for protein synthesis. Reduced translational activity may compromise cell maintenance and remodeling, particularly in neurons undergoing structural plasticity. Furthermore, the downregulation of Lamtor4 and Lamtor5, which are components of the mTOR complex and lysosomal signaling axis [52], points to a suppression of anabolic and growth-related pathways, potentially reflecting a broader metabolic downscaling in PV^+^ cells. Interestingly, this suppression of metabolism-related genes was accompanied by upregulation of transcripts related to intracellular signaling and synaptic function. We found an increased expression of Wnk2 and Ptpn9, which modulate ion transport and signaling cascades [47, 48], and actin-binding genes implicated in cytoskeletal dynamics and synaptic spine plasticity [53]. These shifts may facilitate signal transduction and structural remodeling despite decreased mitochondrial output.

Upregulation of amino acid transporters and potassium channel subunit Kcnf1 suggests enhanced ion flux and neurotransmitter dynamics. Notably, Kcnf1 is known to modulate the gating properties of Kv channels [49], potentially influencing the excitability of PV-INs.

Changes in neuropeptide and synaptic genes further point to alterations in inhibitory tone and synaptic integration. Downregulation of Neuropeptide Y (Npy), which is involved in anxiety regulation and increases inhibitory tone [54], and Parvalbumin (Parv), a calcium-binding protein that ensures fast-spiking fidelity [55, 56], may indicate reduced inhibitory function in PV-INs. In contrast, the upregulation of Synaptotagmin 16 (Syt16), involved in vesicle exocytosis, and Neuronal Pentraxin Receptor (Nptxr), which clusters AMPA receptors at synapses [50], suggests enhanced excitatory input onto PV-INs or altered synaptic integration. This dual regulation may underline complex changes in the excitation-inhibition (E/I) balance following fluoxetine.

We also identified increased expression of genes associated with extracellular matrix remodeling, including Mmp17, a matrix metalloproteinase that degrades perineuronal nets (PNNs), and Ptprs, a CSPG receptor involved in memory retention [16, 51]. These findings suggest active structural remodeling of PNNs in PV-INs in response to fluoxetine, consistent with prior studies implicating PNN modulation in adult cortical plasticity [13, 17].

Taken together, these findings suggest that chronic fluoxetine treatment induces a complex transcriptional profile in PV^+^ cells, characterized by suppression of metabolic and translational capacity alongside enhanced synaptic signaling and structural remodeling. This dual regulation may represent a cellular state shift that enables plasticity at the cost of reduced bioenergetic capacity —consistent with fluoxetine’s known effects on reopening critical period-like plasticity in cortical networks.

### The role of synaptic and circuit level plasticity

Our findings of reduced PV expression and PNN intensity in the PFC following chronic fluoxetine treatment are indicative of a plasticity-permissive state in PV^+^ cells. This interpretation is supported by previous studies demonstrating that chronic fluoxetine leads to increased dendritic spine density and altered expression of plasticity-related markers such as PSA-NCAM and synaptophysin in cortical interneurons [57]. In addition, Alme et al [58]. reported that chronic fluoxetine upregulates BDNF-related LTP-associated genes, including Narp and TIEG1, in the PFC. Moreover, Ohira et al. [59]. provided direct evidence that fluoxetine enhances interneuron plasticity by promoting the generation of new GABAergic neurons from progenitor cells in the adult PFC. These prior observations align with our transcriptomic findings showing upregulation of genes involved in synaptic signaling and cytoskeletal remodeling, such as Syt16, Nptxr, and actin-binding proteins. Collectively, these findings suggest that fluoxetine promotes structural and transcriptional adaptations that enable plasticity in PV^+^ cells

### Fluoxetine-induced modulation of mitochondrial features in PV interneurons

While TOMM22 is a broadly expressed mitochondrial marker, our analysis restricted to PV^+^ cells revealed only a subtle increase in TOMM22 intensity in the PL region following fluoxetine treatment. Across all regions, TOMM22 intensity correlated strongly with PV expression levels, suggesting that mitochondrial content may scale with the functional state or maturity of PV^+^ cells. This observation complements our TRAP-seq data, which revealed robust changes in mitochondrial gene expression following fluoxetine treatment, and together these findings point to fluoxetine-induced modulation of mitochondrial function in PV^+^ cells at both the transcriptional and cellular levels. Further studies using more sensitive protein-level or functional assays may help clarify the functional relevance of these mitochondrial changes.

A major limitation of the ATP and mtDNA assays in this study is that they primarily reflect assays performed on somatic mitochondria. Mitochondria that are localized at axon terminals and synapses, which are critical for PV-INs fast-spiking function, are not adequately captured by these methods. More refined compartment-specific approaches, such as live imaging or proximity-based labeling, will be necessary to resolve these dynamics in future work. The high variability in mitochondrial activity across brain compartments and the rapid turnover of ATP could also mask small but biologically meaningful differences. We tried to determine mitochondrial metabolism rate directly from FACS-sorted PV^+^ cells using a biochemical method (SEAHORSE), but the method was unsuccessful due to scarcity of material obtained.

Despite stable intracellular ATP levels, fluoxetine treatment was associated with transcriptional downregulation of mitochondrial genes in PV^+^ cells, including components of the electron transport chain and mitochondrial protein import machinery. Interestingly, this transcriptional change was accompanied by increased mitochondrial DNA content specifically in PV^+^ cells of the PFC, with a slight but significant increase in TOMM22 signal intensity in the PL region. These findings suggest a potential compensatory mechanism where mitochondrial biogenesis is enhanced to maintain energy homeostasis despite altered gene expression. This selective regulation of mitochondrial features at both transcriptional and DNA levels may influence the energetic capacity and activity-dependent responses of PV-INs, supporting their role in fluoxetine-mediated plasticity, even in the absence of detectable changes in ATP production.

### Potassium channel regulation

Our previous study demonstrated that optical activation of TrkB specifically in PV-INs reduces their excitability via the decreased expression and activity of Kv3.1 [12]. The activation of TrkB via BDNF stimulation similarly modulates excitability and immune responses in the rat olfactory bulb [60]. Conversely, upregulation of Kv3.1b/3.2 channels in response to TrkB ligands BDNF and NT4 have been observed in cultured rat neurons from the visual cortex [61]. These channels are crucial for fast repolarization following an action potential, particularly in fast-spiking interneurons like PV-INs [62]. The differential regulations of the potassium channels observed across these studies likely reflect their complexity, with region- specific roles and developmental, as well as activity-dependent, variations in their expression [63].

Together, the combination of the increased potassium channel gene expression, downregulation of parvalbumin and PNNs, and altered energy dynamics following fluoxetine treatment suggests a change in the inhibitory tone of PV-INs. This shift potentially contributes to the E/I balance alterations towards increased excitation and promoting synaptic plasticity and the remodeling of cortical circuits, which are essential for cognitive flexibility and emotional regulation [64].

### Region-specific modulation of PV-IN plasticity in the prefrontal cortex by chronic fluoxetine treatment

Our study reveals region-specific effects of chronic fluoxetine treatment on PV^+^ cells across key subregions of PFC, aligning with their distinct roles in emotional and cognitive processing. In the PL, associated with fear expression, fluoxetine reduced PV expression, suggesting enhanced plasticity that may aid in emotional regulation and anxiety relief [31, 32]. While no significant changes were observed in the IL, critical for fear extinction, the trend toward reduced inhibitory tone could support the formation of new, less fearful associations [65]. In the ACA/M2, which are involved in cognitive flexibility and motor planning, reduced perineuronal net (PNN) intensity points to increased synaptic remodeling, potentially enhancing adaptability and cognitive function [33]. These findings highlight that fluoxetine’s region-specific enhancement of plasticity in the PFC contribute to its therapeutic effects in treating depression and anxiety by modulating neural circuits related to emotion and cognition.

### Specificity and limitations of the PV-IRES-Cre driver line

In this study, we employed the PV-IRES-Cre line to target parvalbumin-expressing neurons. While this line is widely used for studying PV interneurons, its recombination profile reflects Pvalb promoter activity rather than current PV protein expression. Our validation in the prefrontal cortex demonstrated that 72.8% of tdTomato-positive cells co-expressed PV protein, while only 2.5% showed overlap with SST-positive interneurons (Fig. 4a–c). These values align with previous studies documenting variable recombination efficiency in the PV-IRES-Cre line across brain regions and developmental stages [66]. The incomplete overlap likely reflects cells with a history of PV expression that may have downregulated the protein, as well as the inherent temporal dynamics of Cre-mediated recombination. Recent findings also suggest rare off-target labeling in excitatory neurons [67], emphasizing the importance of region-specific validation. To acknowledge these limitations, we refer to our target population as “PV-positive cells” (PV^+^ cells) rather than strictly “PV interneurons,” reflecting our methodological approach and analytical caution. Despite these caveats, the high specificity for PV lineage (72.8% overlap) and minimal SST cross-reactivity (2.5%) supports the validity of our cell-type-specific findings.

### Clinical implications and future directions

The modulation of PV-INs by fluoxetine carries significant clinical implications, particularly given their crucial role in maintaining cortical network balance. Fluoxetine’s ability to reduce PV expression and PNN density may help restore E/I balance in conditions with pathologically elevated inhibition, offering therapeutic benefits in mood disorders and other neuropsychiatric conditions characterized by disrupted cortical networks [10]. Our findings highlight potential biomarkers related to PV-IN plasticity or mitochondrial activity, which could aid in identifying new therapeutic targets. Future research should focus on exploring the long-term effects of fluoxetine on neural circuitry and cognitive function to better understand its full spectrum of therapeutic outcomes. Additionally, translational research should investigate combining fluoxetine with psychotherapy [2] or treatments targeting complementary pathways, such as synaptic stabilization [68], to enhance its efficacy and broaden its applicability in treating various neuropsychiatric disorders.

## Supplementary information

Supplemental note

Supplemental Table 1

Supplemental Table 2

Supplemental Table 3

Supplemental Table 4

## Data Availability

The RNA sequencing data will be deposited in a public repository (Gene Expression Omnibus, GEO) upon publication. Raw and processed RNA sequencing data files from the TRAP experiments, including differential gene expression results and pathway analyses, are available from the corresponding authors upon reasonable request. Analysis scripts used for processing the TRAP-seq data, FACS analyses, and immunohistochemical quantification are available from the corresponding authors upon request. The datasets generated and analyzed during the current study, including raw microscopy images, FACS data files, and quantification spreadsheets, are available from the corresponding author on reasonable request. Mouse lines used in this study are available from Jackson Laboratory (PV-IRES-Cre, stock #008069; Ai9, stock #007909).

## References

[1] Castrén E, Hen R. Neuronal plasticity and antidepressant actions. Trends Neurosci. 2013;36:259–67. 23380665 10.1016/j.tins.2012.12.010 PMC3648595

[2] Umemori J, Winkel F, Didio G, Llach Pou M, Castrén E. iPlasticity: Induced juvenile-like plasticity in the adult brain as a mechanism of antidepressants. Psychiatry Clin Neurosci. 2018;72:633–53. 29802758 10.1111/pcn.12683 PMC6174980

[3] Castrén E. Is mood chemistry?. Nat Rev Neurosci. 2005;6:241–6. 15738959 10.1038/nrn1629

[4] Vetencourt JFM, Sale A, Viegi A, Baroncelli L, De Pasquale R, O’Leary OF, et al. The antidepressant fluoxetine restores plasticity in the adult visual cortex. Science. 2008;320:385–8. 18420937 10.1126/science.1150516

[5] Malberg JE, Duman RS. Cell proliferation in adult hippocampus is decreased by inescapable stress: reversal by fluoxetine treatment. Neuropsychopharmacology. 2003;28:1562–71. 12838272 10.1038/sj.npp.1300234

[6] Karpova NN, Pickenhagen A, Lindholm J, Tiraboschi E, Kulesskaya N, Ágústsdóttir A, et al. Fear erasure in mice requires synergy between antidepressant drugs and extinction training. Science. 2011;334:1731–4. 22194582 10.1126/science.1214592 PMC3929964

[7] Jetsonen E, Didio G, Winkel F, Llach Pou M, Boj C, Kuczynski-Noyau L, et al. Activation of TrkB in Parvalbumin interneurons is required for the promotion of reversal learning in spatial and fear memory by antidepressants. Neuropsychopharmacology. 2023;48:1021–30. 10.1038/s41386-023-01562-y PMC10209093 36944718

[8] Casarotto PC, Girych M, Fred SM, Kovaleva V, Moliner R, Enkavi G, et al. Antidepressant drugs act by directly binding to TRKB neurotrophin receptors. Cell. 2021;184:1299–1313.e19. 33606976 10.1016/j.cell.2021.01.034 PMC7938888

[9] Bartos M, Vida I, Jonas P. Synaptic mechanisms of synchronized gamma oscillations in inhibitory interneuron networks. Nat Rev Neurosci. 2007;8:45–56. 17180162 10.1038/nrn2044

[10] Sohal VS, Zhang F, Yizhar O, Deisseroth K. Parvalbumin neurons and gamma rhythms enhance cortical circuit performance. Nature. 2009;459:698–702. 19396159 10.1038/nature07991 PMC3969859

[11] Donato F, Rompani SB, Caroni P. Parvalbumin-expressing basket-cell network plasticity induced by experience regulates adult learning. Nature. 2013;504:272–6. 24336286 10.1038/nature12866

[12] Winkel F, Ryazantseva M, Voigt MB, Didio G, Lilja A, Llach Pou M, et al. Pharmacological and optical activation of TrkB in Parvalbumin interneurons regulate intrinsic states to orchestrate cortical plasticity. Mol Psychiatry. 2021;26:7247–56. 34321594 10.1038/s41380-021-01211-0 PMC8872988

[13] Fawcett JW, Oohashi T, Pizzorusso T. The roles of perineuronal nets and the perinodal extracellular matrix in neuronal function. Nat Rev Neurosci. 2019;20:451–65. 31263252 10.1038/s41583-019-0196-3

[14] Kwok JCF, Dick G, Wang D, Fawcett JW. Extracellular matrix and perineuronal nets in CNS repair. Dev Neurobiol. 2011;71:1073–89. 21898855 10.1002/dneu.20974

[15] Frischknecht R, Heine M, Perrais D, Seidenbecher CI, Choquet D, Gundelfinger ED. Brain extracellular matrix affects AMPA receptor lateral mobility and short-term synaptic plasticity. Nat Neurosci. 2009;12:897–904. 19483686 10.1038/nn.2338

[16] Lesnikova A, Casarotto P, Moliner R, Fred SM, Biojone C, Castrén E. Perineuronal net receptor PTPσ regulates retention of memories. Front Synaptic Neurosci. 2021;13:37. 10.3389/fnsyn.2021.672475 PMC8339997 34366821

[17] Tewari BP, Chaunsali L, Campbell SL, Patel DC, Goode AE, Sontheimer H. Perineuronal nets decrease membrane capacitance of peritumoral fast spiking interneurons in a model of epilepsy. Nat Commun. 2018;9:4724. 30413686 10.1038/s41467-018-07113-0 PMC6226462

[18] Alaiyed S, McCann M, Mahajan G, Rajkowska G, Stockmeier CA, Kellar KJ, et al. Venlafaxine Stimulates an MMP-9-Dependent increase in excitatory/inhibitory balance in a stress model of depression. J Neurosci. 2020;40:4418–31. 32269106 10.1523/JNEUROSCI.2387-19.2020 PMC7252486

[19] Kann O, Papageorgiou IE, Draguhn A. Highly energized inhibitory interneurons are a central element for information processing in cortical networks. J Cereb Blood Flow Metab. 2014;34:1270–82. 24896567 10.1038/jcbfm.2014.104 PMC4126088

[20] Faria-Pereira A, Morais VA. Synapses: The Brain’s Energy-Demanding Sites. Int J Mol Sci. 2022;23:3627. 35408993 10.3390/ijms23073627 PMC8998888

[21] Cheng A, Hou Y, Mattson MP. Mitochondria and neuroplasticity. ASN Neuro. 2010;2:AN20100019. 10.1042/AN20100019 PMC2949087 20957078

[22] Lee A, Hirabayashi Y, Kwon S-K, Lewis TL, Polleux F. Emerging roles of mitochondria in synaptic transmission and neurodegeneration. Curr Opin Physiol. 2018;3:82–93. 30320242 10.1016/j.cophys.2018.03.009 PMC6178220

[23] Calabresi P, Gubellini P, Picconi B, Centonze D, Pisani A, Bonsi P, et al. Inhibition of Mitochondrial Complex II Induces a Long-Term Potentiation of NMDA-Mediated Synaptic Excitation in the Striatum Requiring Endogenous Dopamine. Journal Neurosci. 2001;21:5110–20. 10.1523/JNEUROSCI.21-14-05110.2001 PMC6762835 11438586

[24] Filipović D, Costina V, Perić I, Stanisavljević A, Findeisen P. Chronic fluoxetine treatment directs energy metabolism towards the citric acid cycle and oxidative phosphorylation in rat hippocampal nonsynaptic mitochondria. Brain Res. 2017;1659:41–54. 28119059 10.1016/j.brainres.2017.01.025

[25] Curti C, Mingattao FE, Polizello ACM, Galastri LO, Uyemura SA, Santos AC. Fluoxetine interacts with the lipid bilayer of the inner membrane in isolated rat brain mitochondria, inhibiting electron transport and F1F0-ATPase activity. Mol Cell Biochem. 1999;199:103–9. 10544958 10.1023/a:1006912010550

[26] Hroudová J, Fišar Z. In vitro inhibition of mitochondrial respiratory rate by antidepressants. Toxicol Lett. 2012;213:345–52. 22842584 10.1016/j.toxlet.2012.07.017

[27] Agostinho FR, Réus GZ, Stringari RB, Ribeiro KF, Ferreira GK, Jeremias IC, et al. Olanzapine plus fluoxetine treatment alters mitochondrial respiratory chain activity in the rat brain. Acta Neuropsychiatr. 2011;23:282–91. 25380039 10.1111/j.1601-5215.2011.00569.x

[28] Miller EK, Cohen JD. An integrative theory of prefrontal cortex function. Annu Rev Neurosci. 2001;24:167–202. 11283309 10.1146/annurev.neuro.24.1.167

[29] Grace AA, Floresco SB, Goto Y, Lodge DJ. Regulation of firing of dopaminergic neurons and control of goal-directed behaviors. Trends Neurosci. 2007;30:220–7. 17400299 10.1016/j.tins.2007.03.003

[30] Guirado R, Perez-Rando M, Ferragud A, Gutierrez-Castellanos N, Umemori J, Carceller H, et al. A critical period for prefrontal network configurations underlying psychiatric disorders and addiction. Front Behav Neurosci. 2020;14:51. 10.3389/fnbeh.2020.00051 PMC7155216 32317945

[31] Sotres-Bayon F, Cain CK, LeDoux JE. Brain mechanisms of fear extinction: historical perspectives on the contribution of prefrontal cortex. Biol Psychiatry. 2006;60:329–36. 16412988 10.1016/j.biopsych.2005.10.012

[32] Quirk GJ, Mueller D. Neural Mechanisms of Extinction Learning and Retrieval. Neuropsychopharmacology. 2008;33:56–72. 17882236 10.1038/sj.npp.1301555 PMC2668714

[33] Bush G, Luu P, Posner MI. Cognitive and emotional influences in anterior cingulate cortex. Trends Cogn Sci. 2000;4:215–22. 10827444 10.1016/s1364-6613(00)01483-2

[34] Arnone D. Functional MRI findings, pharmacological treatment in major depression and clinical response. Prog Neuropsychopharmacol Biol Psychiatry. 2019;91:28–37. 30099082 10.1016/j.pnpbp.2018.08.004

[35] Hippenmeyer S, Vrieseling E, Sigrist M, Portmann T, Laengle C, Ladle DR, et al. A Developmental Switch in the Response of DRG Neurons to ETS Transcription Factor Signaling. PLoS Biol. 2005;3:e159. 15836427 10.1371/journal.pbio.0030159 PMC1084331

[36] Liu J, Krautzberger AM, Sui SH, Hofmann OM, Chen Y, Baetscher M, et al. Cell-specific translational profiling in acute kidney injury. J Clin Invest. 2014;124:1242–54. 24569379 10.1172/JCI72126 PMC3938273

[37] Madisen L, Zwingman TA, Sunkin SM, Oh SW, Zariwala HA, Gu H, et al. A robust and high-throughput Cre reporting and characterization system for the whole mouse brain. Nat Neurosci. 2010;13:133–40. 20023653 10.1038/nn.2467 PMC2840225

[38] Heiman M, Kulicke R, Fenster RJ, Greengard P, Heintz N. Cell type-specific mRNA purification by translating ribosome affinity purification (TRAP). Nat Protoc. 2014;9:1282–91. 24810037 10.1038/nprot.2014.085 PMC4102313

[39] Ting JT, Daigle TL, Chen Q, Feng G. Acute Brain Slice Methods for Adult and Aging Animals: Application of Targeted Patch Clamp Analysis and Optogenetics 2014. p. 221-42. 10.1007/978-1-4939-1096-0_14 PMC4219416 25023312

[40] George Paxinos, Keith B. J. Franklin. The mouse brain in stereotaxic coordinates, 2nd edition. Academic Press; 2001.

[41] Love MI, Huber W, Anders S. Moderated estimation of fold change and dispersion for RNA-seq data with DESeq2. Genome Biol. 2014;15:1–21. 10.1186/s13059-014-0550-8 PMC4302049 25516281

[42] Wu T, Hu E, Xu S, Chen M, Guo P, Dai Z, et al. clusterProfiler 4.0: A universal enrichment tool for interpreting omics data. Innovation. 2021;2:100141. 34557778 10.1016/j.xinn.2021.100141 PMC8454663

[43] den Brave F, Pfanner N, Becker T. Mitochondrial entry gate as regulatory hub. Biochimica et Biophys Acta (BBA) - Mol Cell Res. 2024;1871:119529. 10.1016/j.bbamcr.2023.119529 37951505

[44] Sousa JS, D’Imprima E, Vonck J. Mitochondrial Respiratory Chain Complexes 2018. p. 167–227. 10.1007/978-981-10-7757-9_7 29464561

[45] Vanden Broeck A, Klinge S. Eukaryotic Ribosome Assembly. Annu Rev Biochem. 2024;93:189–210. 38768392 10.1146/annurev-biochem-030222-113611

[46] Campos RK, Wijeratne HRS, Shah P, Garcia-Blanco MA, Bradrick SS. Ribosomal stalk proteins RPLP1 and RPLP2 promote biogenesis of flaviviral and cellular multi-pass transmembrane proteins. Nucleic Acids Res. 2020;48:9872–85. 32890404 10.1093/nar/gkaa717 PMC7515724

[47] Tiganis T, Bennett AM. Protein tyrosine phosphatase function: the substrate perspective. Biochem J. 2007;402:1–15. 17238862 10.1042/BJ20061548 PMC1783993

[48] Rinehart J, Vázquez N, Kahle KT, Hodson CA, Ring AM, Gulcicek EE, et al. WNK2 Kinase Is a novel regulator of essential neuronal cation-chloride cotransporters. J Biol Chem. 2011;286:30171–80. 21733846 10.1074/jbc.M111.222893 PMC3191056

[49] Kramer JW, Post MA, Brown AM, Kirsch GE. Modulation of potassium channel gating by coexpression of Kv2.1 with regulatory Kv5.1 or Kv6.1 α-subunits. Am J Physiol-Cell Physiol. 1998;274:C1501–C1510. 10.1152/ajpcell.1998.274.6.C1501 9696692

[50] Pelkey KA, Barksdale E, Craig MT, Yuan X, Sukumaran M, Vargish GA, et al. Pentraxins coordinate excitatory synapse maturation and circuit integration of parvalbumin interneurons. Neuron. 2015;85:1257–72. 25754824 10.1016/j.neuron.2015.02.020 PMC4368480

[51] Alaiyed S, Conant K. A Role for matrix metalloproteases in antidepressant efficacy. Front Mol Neurosci. 2019;12:1–10. 10.3389/fnmol.2019.00117 PMC6517485 31133801

[52] Bar-Peled L, Sabatini DM. Regulation of mTORC1 by amino acids. Trends Cell Biol. 2014;24:400–6. 24698685 10.1016/j.tcb.2014.03.003 PMC4074565

[53] Borovac J, Bosch M, Okamoto K. Regulation of actin dynamics during structural plasticity of dendritic spines: Signaling messengers and actin-binding proteins. Mol Cell Neurosci. 2018;91:122–30. 30004015 10.1016/j.mcn.2018.07.001

[54] Tasan RO, Verma D, Herzog H, Sperk G. Neuropeptide Y in the basolateral amygdala modulates the acquisition of conditioned fear. BMC Pharm. 2010;10:A33.

[55] Caillard O, Moreno H, Schwaller B, Llano I, Celio MR, Marty A. Role of the calcium-binding protein parvalbumin in short-term synaptic plasticity. Proc Natl Acad Sci. 2000;97:13372–7. 11069288 10.1073/pnas.230362997 PMC27231

[56] Schwaller B. The continuing disappearance of “pure” Ca2+ buffers. Cellular Mol Life Sci. 2009;66:275–300. 19099190 10.1007/s00018-008-8564-6 PMC11131537

[57] Guirado R, Perez-Rando M, Sanchez-Matarredona D, Castrén E, Nacher J. Chronic fluoxetine treatment alters the structure, connectivity and plasticity of cortical interneurons. Int J Neuropsychopharmacol. 2014;17:1635–46. 24786752 10.1017/S1461145714000406

[58] Alme MN, Wibrand K, Dagestad G, Bramham CR. Chronic fluoxetine treatment induces brain region-specific upregulation of genes associated with BDNF-induced long-term potentiation. Neural Plast. 2007;2007:1–9. 10.1155/2007/26496 PMC2248427 18301726

[59] Ohira K, Takeuchi R, Shoji H, Miyakawa T. Fluoxetine-Induced Cortical Adult Neurogenesis. Neuropsychopharmacology. 2013;38:909–20. 23303069 10.1038/npp.2013.2 PMC3629401

[60] Tucker K, Fadool DA. Neurotrophin modulation of voltage-gated potassium channels in rat through TrkB receptors is time and sensory experience dependent. J Physiol. 2002;542:413–29. 12122142 10.1113/jphysiol.2002.017376 PMC2290412

[61] Grabert J, Wahle P. Neuronal activity and TrkB ligands influence Kv3.1b and Kv3.2 expression in developing cortical interneurons. Neuroscience. 2008;156:618–29. 18775767 10.1016/j.neuroscience.2008.08.008

[62] RUDY B, CHOW A, LAU D, AMARILLO Y, OZAITA A, SAGANICH M, et al. Contributions of Kv3 Channels to Neuronal Excitability. Ann N Y Acad Sci. 1999;868:304–43. 10414303 10.1111/j.1749-6632.1999.tb11295.x

[63] Johnston J, Forsythe ID, Kopp-Scheinpflug C. SYMPOSIUM REVIEW: Going native: voltage-gated potassium channels controlling neuronal excitability. J Physiol. 2010;588:3187–200. 20519310 10.1113/jphysiol.2010.191973 PMC2976014

[64] Ferguson BR, Gao W-J. PV Interneurons: Critical regulators of E/I Balance for prefrontal cortex-dependent behavior and psychiatric disorders. Front Neural Circuits. 2018;12:37. 10.3389/fncir.2018.00037 PMC5964203 29867371

[65] Milad MR, Quirk GJ. Fear Extinction as a model for translational neuroscience: ten years of progress. Annu Rev Psychol. 2012;63:129–51. 22129456 10.1146/annurev.psych.121208.131631 PMC4942586

[66] Nigro MJ, Kirikae H, Kjelsberg K, Nair RR, Witter MP. Not All That Is Gold Glitters: PV-IRES-Cre Mouse Line shows low efficiency of labeling of parvalbumin interneurons in the perirhinal cortex. Front Neural Circuits. 2021;15:1–9. 10.3389/fncir.2021.781928 PMC8606682 34819840

[67] Palicz R, Pater B, Truschow P, Witte M, Staiger JF. Intersectional strategy to study cortical inhibitory parvalbumin-expressing interneurons. Sci Rep. 2024;14:2829. 38310185 10.1038/s41598-024-52901-y PMC10838283

[68] Duman RS, Sanacora G, Krystal JH. Altered connectivity in depression: GABA and glutamate neurotransmitter deficits and reversal by novel treatments. Neuron. 2019;102:75–90. 30946828 10.1016/j.neuron.2019.03.013 PMC6450409

