# Supplementary material for: Chronic treatment with fluoxetine regulates mitochondrial features and plasticity-associated transcriptomic pathways in parvalbumin-positive interneurons of prefrontal cortex
Source: Neuropsychopharmacology. 2025 Sep 8;50(12):1864–74. doi: 10.1038/s41386-025-02219-8 (PMC12518560; doi:10.1038/s41386-025-02219-8)
Supplement: Supplementary file 1 — Supplemental note [file 41386_2025_2219_MOESM1_ESM.docx]

**Supplemental note**

## **Primers used for mtDNA detection**

mDNA-MT-Rnr1-F AGGAGCCTGTTCTATAATCGATAAA

mDNA-MT-Rnr1-R GATGGCGGTATATAGGCTGAA

mDNA-Rbm15-F GGACACTTTTCTTGGGCAAC

mDNA-Rbm15-R AGTTTGGCCCTGTGAGACAT

**Supplemental figure 1**

Fluoxetine consumption was calculated by measuring the weight of each water bottle before and after being available for mice twice per week. a) Daily water consumption for mice in fluoxetine and control group. b) Daily fluoxetine consumption for each mouse in the fluoxetine treated group. Average consumption was 31.0 mg/kg/day.

**Supplemental figure 2**

**RNA integrity**


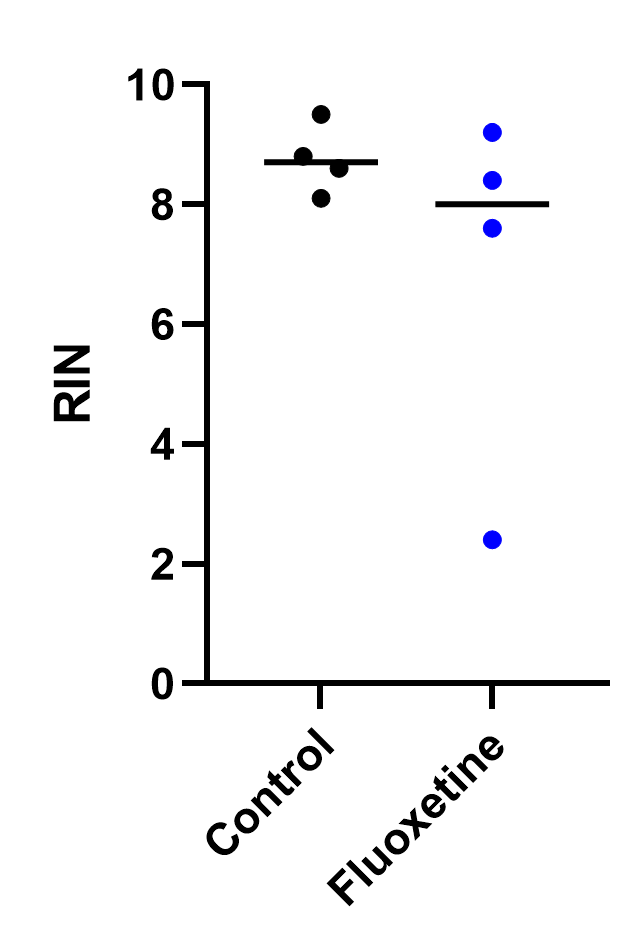


RNA Integrity Check. RNA quality was assessed using RNA integrity number (RIN) measurements obtained from Bioanalyzer (Agilent, California, US). One sample from the fluoxetine-treated group showed a low RIN value of 2.4 (marked with a red 'x') and was excluded from further analyses. The RIN values for all other samples ranged between 7.6 and 9.5, indicating good RNA integrity.

**Supplemental figure 3**


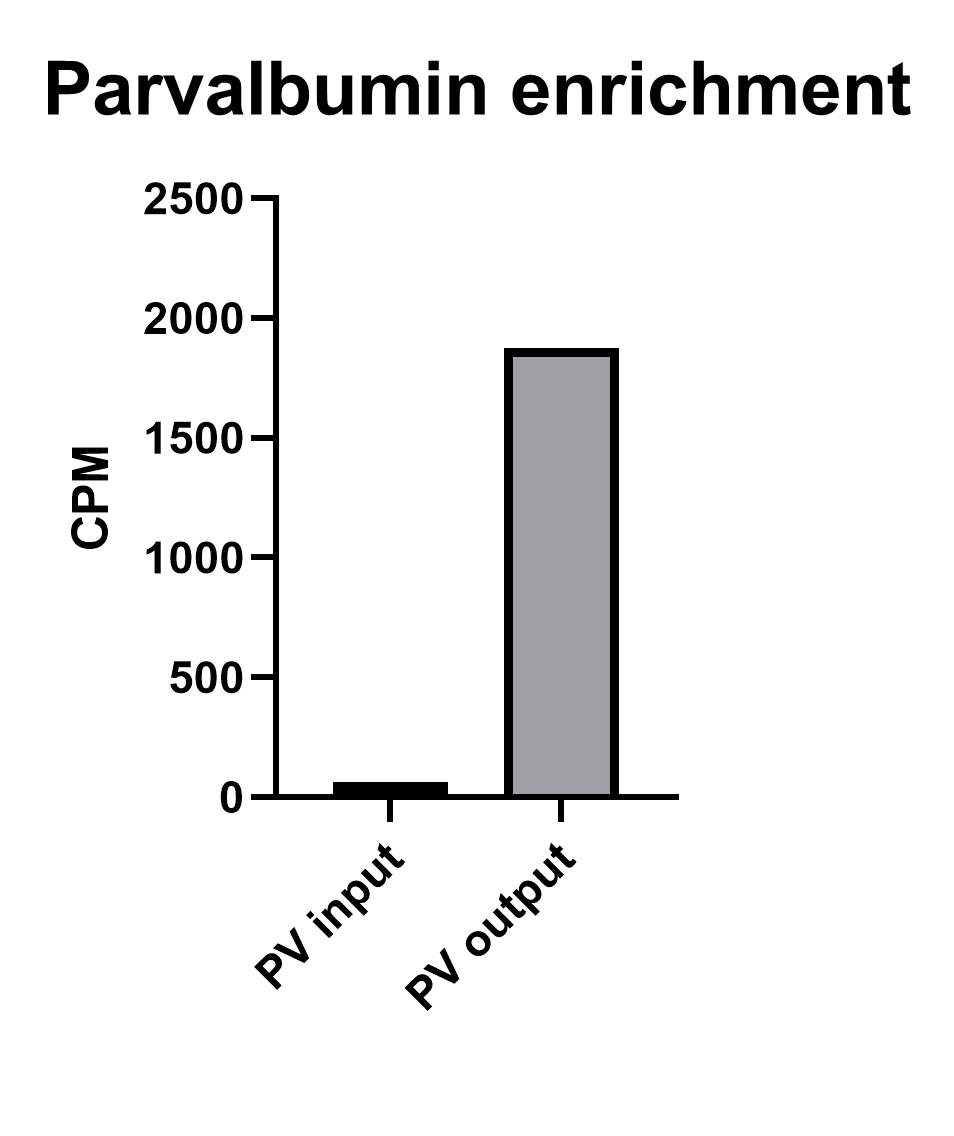


Preliminary Validation of TRAP Immunoprecipitation for PV-INs. Counts per million (CPM) of Parvalbumin mRNA were measured before and after TRAP analysis (N = 2 for pre-TRAP, N = 7 for post-TRAP). The results indicate an enrichment of Parvalbumin mRNA following TRAP, providing a preliminary confirmation that RNA from PV-INs was successfully enriched during the immunoprecipitation process.

**Supplemental figure 4**
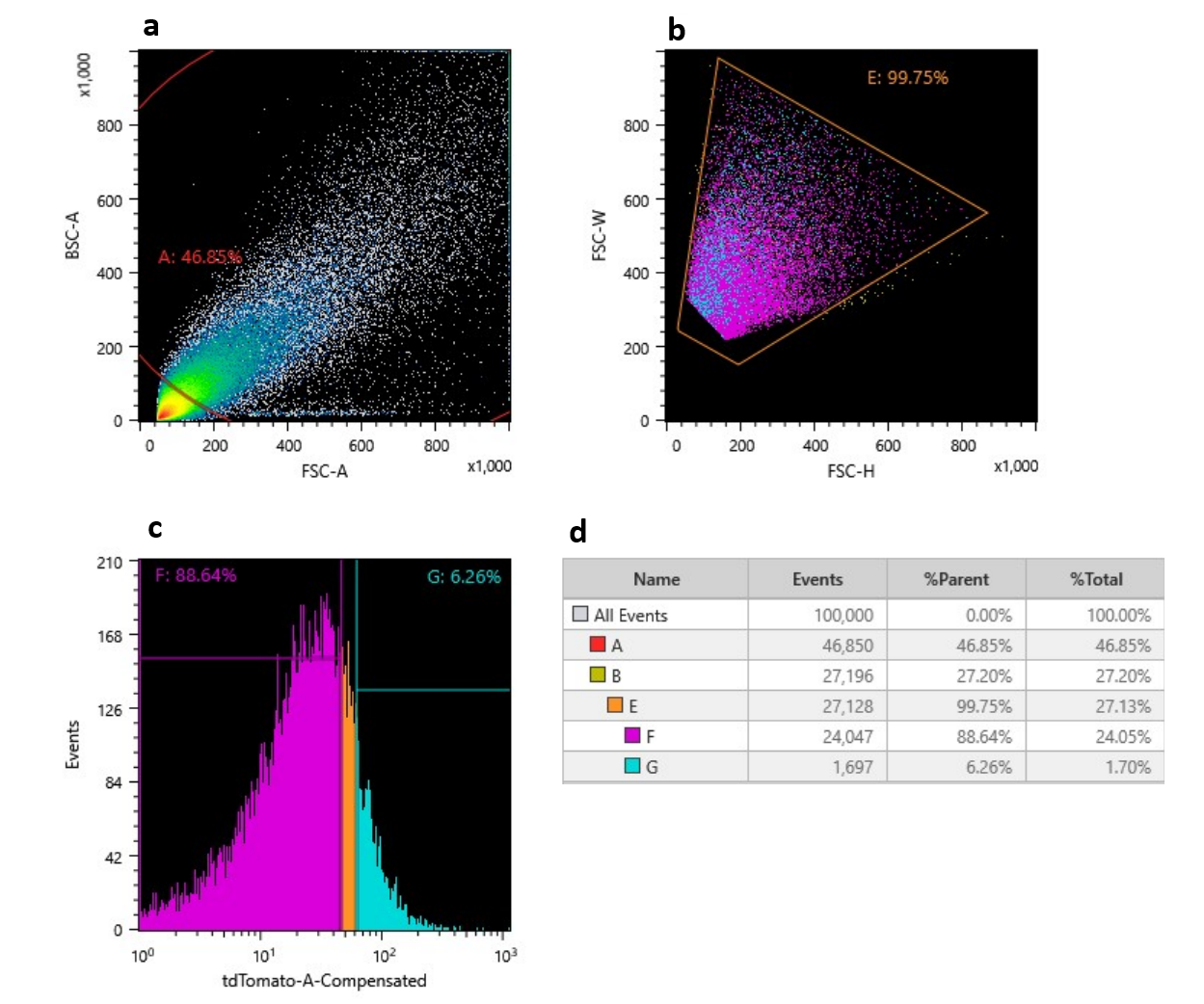


Gating strategy for FACS-based sorting. (a) All detected cell events were first gated using backward and forward scatter area (Gate A) to exclude debris. (b) Further debris exclusion was made using forward scatter area and width. Cells in Gate E were selected for further sorting. (c) TdTomato-positive cells were identified based on their fluorescent intensity. Cells within Gate G were sorted as PV+ cells, and those in Gate F represent were collected as “Non-PV-INs” (c) Representative percentage of cells in different groups out of 100 000 initially detected particles. G represents PV+ cells, and G non-PV cells.

Supplemental table 1

Antibodies used in all experiments.

Supplemental table 2

All detected DEGs with annotation information from DAVID database. The average expression of each group (control or fluoxetine) was measured in counts per million (CPM). Q-value cutoff < 0.1 was used.

Supplemental table 3

All detected Gene Ontology Molecular Function pathways from pathway analysis. Up- and downregulated pathways were analyzed separately. Column “p.adjust” is p-value adjusted with Benjamini-Hochberg method and “qvalue” is p-value adjusted with FDR.

Supplemental table 4

Statistical tests used for FACS and IHC experiments.
